# Influenza Virus Genomic Mutations, Host Barrier and Cross-species Transmission

**DOI:** 10.2174/0113892029316603240926051325

**Published:** 2024-10-11

**Authors:** Wenyan Xiong, Zongde Zhang

**Affiliations:** 1 Inflammation & Allergic Diseases Research Unit, Affiliated Hospital of Southwest Medical University, Luzhou, Sichuan, 646000, China;; 2 The School of Basic Medical Sciences, Southwest Medical University, Luzhou, Sichuan, 646000, China

**Keywords:** Influenza, host barrier, cross-species transmission, sialic acid, microRNA, microRNA recognition elements

## Abstract

Influenza is a global epidemic infectious disease that causes a significant number of illnesses and deaths annually. Influenza exhibits high variability and infectivity, constantly jumping from birds to mammals. Genomic mutations of the influenza virus are a central mechanism leading to viral variation and antigenic evolution. Amino acid substitutions and avoidance of microRNA recognition elements are crucial in facilitating the virus to cross species barriers. This review summarizes the types of genomic mutations in the influenza virus, their roles and mechanisms in crossing species barriers, and analyzes the impact of these mutations on human health.

## 1 INTRODUCTION

Influenza virus, an RNA virus belonging to the family Orthomyxoviridae, possesses a segmented genome consisting of eight single-stranded negative-sense RNA molecules. Each RNA segment encodes multiple proteins, including the surface proteins hemagglutinin (HA) and neuraminidase (NA), the viral RNA polymerases PB1, PB2, and PA, the matrix protein (M) involved in virus structure, the nucleoprotein (NP) forming the viral core, and the non-structural protein (NS). During synthesis, the HA protein is produced as a precursor called HA0, which subsequently undergoes proteolytic cleavage at a conserved arginine residue into HA1 and HA2 subunits. This cleavage process is essential for the infectivity of the virus. Viruses carrying highly cleavable HAs can cause a rapidly fatal multisystemic infection in poultry, known as Highly Pathogenic Avian Influenza (HPAI), whereas avian IAVs lacking multiple basic amino acid sites are referred to as Low Pathogenic Avian Influenza (LPAI) [1]. Highly Pathogenic Influenza Viruses (HPIV), such as H5N1 and H7N9, typically induce severe respiratory diseases with high morbidity and mortality rates when transmitted from birds or other animals to humans, posing a significant threat to human health.

On the other hand, Low Pathogenic Influenza Virus subtypes (LPIV), like H1N1 and H3N2, generally cause mild symptoms resembling the common cold, including fever, cough, sneezing, headache, and nasal congestion, but usually do not lead to severe complications or death. Genomic mutations can transform low-pathogenic influenza viruses into highly pathogenic ones, triggering global pandemics and impacting influenza viruses' antigenicity, transmissibility, and treatment strategies. Understanding the types and mechanisms of genomic mutations in influenza viruses is crucial for developing preventive and control measures.

## 2 THE BASIC STRUCTURE OF THE INFLUENZA VIRUS AND ITS MUTATIONS

High pathogenic influenza virus often causes severe immune damage to the host, possibly due to enhanced viral genome replication and strong activation of the immune response at the transcriptional level [2]. PB1 protein is a core component of the influenza virus, playing a crucial role in viral replication and transcription processes. As part of the RNA polymerase complex along with PB2 and PA proteins. Studies have shown that crucial amino acid residues and motifs within PB1-F2 can influence the virulence of Influenza A Virus (IAV) in a strain- and host-specific manner by inducing apoptosis, regulating type I IFN response, activating inflammasomes, and promoting secondary bacterial infections [3].

Interactions between PB1 protein and other viral proteins facilitate viral genome replication and transcription within host cells, thereby enhancing viral replication and spread. MAVS is a protein involved in immune responses critical to cellular signaling. Research suggests that binding of PB1-F2 to MAVS protein may lead to MAVS sequestration and inactivation, potentially affecting the immune system's response to the virus. Moreover, studies indicate that the N66S mutation in PB1-F2 enhances its affinity for MAVS binding, leading to more effective suppression of RIG-I-dependent type I Interferon (IFN) production by dissipating mitochondrial transmembrane potential [4, 5]. Given the significant role of PB1 protein in the lifecycle of the influenza virus, it serves as an essential target for studying antiviral drugs and vaccine development. Transcriptomic analysis has revealed that the N66S mutation in PB1-F2 inhibits early type I IFN response but exacerbates late-stage IFN response during IAV infection [6, 7]. The delayed induction of type I IFN by PB1-F2 with the S66 mutation enhances viral lung titers and exacerbates pulmonary immunopathology in IAV-infected mice [8]. Additionally, mutations such as PB1-Q621R have been associated with increased viral replication and transmission in piglets [9]. A study that introduced mutations I298L, K386R, and V517I in the PB1 gene observed reduced viral growth and decreased hemagglutination titers and neuraminidase activity compared to the wild- type virus [10].

The PB2 protein is crucial in the influenza virus. Common mutations at the 627th amino acid position include E (glutamic acid) and K (lysine). This mutation can affect the pathogenicity and adaptability of the influenza virus. Generally, the PB2 protein with the 627E mutation is commonly associated with avian influenza viruses, while the PB2 protein with the 627K mutation is typically associated with human influenza viruses. The 627K mutation can enhance the replication capability and adaptability of the influenza virus in human respiratory tract cells, thereby increasing the risk of human-to-human transmission. For example, the H5N1 avian influenza virus possesses this mutation, enabling it to infect humans [11]. Isolate CS/1000 of the human H3N8 virus has the PB2-E627V substitution [12], which is related to the adaptability of the H5N1 virus, enhanced polymerase activity of the H9N2 virus, and increased toxicity to mice. Studies have indicated that in birds and mammals, PB2-627V exhibits transmissibility and stability [13-15]. In the PB2 protein of HPAI H7N9, the substitutions T271A, Q591K, E627K, and D701N are associated with increased polymerase activity and enhanced toxicity in mice [16, 17]. Additionally, in the PB2 protein of the H10N3 virus, Glu(Glutamate) is detected at the 627th position, indicating avian replicative characteristics without an increase in pathogenicity.

Furthermore, the 701 Asp (Aspartic Acid)and 702 Lys (Lysine)in the PB2 protein display avian replicative preferences [17]. Several replacement mutations, such as PB2-I292V, PB2-R389K, and PB2-A588V, have significantly increased in recent years. Among these, PB2-A588V is considered a key mutation site for co-adaptation to birds and mammals [18].

Moreover, the PB2-D195N in LPAI H9N2 notably increases polymerase activity in mammalian cells [19]. The virulence of the H10N4 virus exhibits a 40% mortality rate in non-pre-adapted mice, and molecular analysis indicates the presence of the PB2-D195N mutation. Further research must understand the relationship between PB2-D195N substitution and mouse pathogenicity [20]. For H3N2, the residues encoded in the PB2 gene: 271A, 590S, 591R, and 661A, as well as the 669V residue encoded in the PA gene, support the viewpoint that these swine viruses represent an increased risk of zoonotic transmission [21-24].

Hemagglutinin (HA) and Neuraminidase (NA) are two crucial surface proteins of the influenza virus. It is generally recognized that there are 16 HA and 9 subtypes of NA. Recently, new subtypes H17, H18, N10, and N11 have been identified in bats, leading to the classification of influenza- like viruses [25].

HA, also known as hemagglutinin, is a glycoprotein on the surface of the influenza virus with functions related to blood clotting and viral invasion of host cells. HA plays a vital role in the virus-host cell interaction by binding to specific receptors on the host cell surface, facilitating viral attachment and entry into the host cell through endocytosis. Studies on the Eurasian avian-like H1N1 swine flu virus have shown that a glycine (G) to glutamic acid (E) substitution at amino acid position 225 in the HA1 protein accelerates virus assembly and budding, enhancing the spread of the virus in guinea pigs [26]. A double amino acid change, D190E, and D225G, in the HA protein also abolish the 1918/H1N1 influenza virus transmission in ferrets [27]. Amino acid residues 222 and 226 in the HA protein are crucial for binding the 2009/H1N1 virus to human receptors and inter-mammalian transmission [28, 29].

The HA protein's membrane-binding site (MBS) is critical for the influenza virus to enter host cells. Studies on highly pathogenic H5N1 found that infection of endothelial cells by H5N1 leads to excessive cytokine production, compromising lung endothelial barrier integrity and causing vascular leakage and viral pneumonia. The MBS in HA is essential for the endothelial cell-mediated pathogenesis of H5N1 [30]. Mutations at positions 158 or 160 results in the loss of N-glycosylation sites at HA positions 158-160, affecting receptor binding specificity and the virulence of HPAI H5 viruses [31].

The stability of HA has been shown to promote adaptation in humans and ferrets by aiding virus particles in resisting inactivation in mildly acidic environments, allowing them to trigger both early and late endosomal membrane fusion [32]. Compared to wild-type viruses with an HA activation pH of 5.6-6.0, those with a stable pH protein exhibit more excellent fitness, virulence, and transmissibility in chickens and wild ducks. An HA activation pH above 5.5 supports replication and transmission in avian hosts, while a pH of 5.5 or below favors upper respiratory tract replication and airborne transmission in ferrets. Interestingly, opposite effects were observed regarding viral replication and pathogenicity in the lungs [33]. The A/H1N1/2009 HA protein has an activation pH of 6.0, a relatively high value typically associated with avian influenza viruses, facilitating a more muscular type I IFN response by accelerating infection of dendritic cells [34]. In H7N9 HA, the A125T + A151T + L217Q mutation enhances the stability of the virus. The A125T and A151T double mutations introduce glycosylation at amino acids 123 and 149, reducing the thermal stability of the virus HA, while L217Q significantly improves the thermal stability of the virus HA [35].

Neuraminidase (NA) is a glycoprotein on the surface of influenza viruses, playing a crucial role in the late stages of the virus replication cycle. NA cleaves the bonds between sugar molecules on the virus and host cell surfaces, facilitating the release of newly formed viruses and promoting viral spread. Additionally, NA helps prevent virus aggregation on cell surfaces, thereby avoiding recognition and clearance by the host immune system. NA is involved in the regulation of CD83, contributing to hypercytokinemia. Treatment targeting CD83 can alleviate lung damage induced by the influenza virus in mice, offering a novel approach for preventing and treating lung injury caused by the influenza virus [36].

Hemagglutinin (HA) and NA are vital factors in the infection and transmission of influenza viruses. HA mediates virus entry into host cells, while NA is involved in virus release and spread. They are integral components of influenza virus antigens and crucial targets for vaccine development, prevention, and treatment. Antibodies targeting HA and NA can neutralize the virus, blocking its invasion and transmission, thus providing immunity against the influenza virus. The balance between HA and NA is essential for virus generation, host adaptation, and interspecies transmission [37-39]. The higher binding ability of HA to receptors can enhance the cleavage efficiency of NA [40]. However, elevated activity or inhibition of HA or NA can disrupt the HA-NA balance, leading to mutations in both proteins [41-43]. Research has identified HA mutations induced by NA antibody pressure, where increased NA antibody pressure enhances mutations at positions 166, 198, and 234 in H9N2 virus HA. Mutants containing Q234 and N166 exhibit a higher affinity for avian receptors but a weaker affinity for human receptors. Glycosylation at position 166 (corresponding to position 158 in H3 numbering) can enhance the pathogenicity of H5N1 and H5N6 viruses in mice [44]. In a report from 2019, the presence of the I117T point mutation was identified in the NA gene segment of SKH13N6, indicating a genetic variation at that position [45].

Regarding NS1 protein, H5N8-B subclade 2.3.4.4 viruses prefer a shorter NS1 with a truncated C-terminus, resembling human influenza viruses and AIVs infecting humans and animals. H5N8 viruses carrying the truncated NS1 show higher intercellular transmission capability, more effectively inhibit IFN-β response, and induce less apoptosis, with minimal impact on viral replication in human lung cells. Truncation of NS1 also increases the virulence of H5N8 in mice, regardless of the virus backbone, making it a virulence marker for H5N8-like viruses in mammals [46]. The single amino acid mutation P42S in the NS1 gene of H5N1 (A/Duck/ Guangxi/12/03) has been linked to increased virulence in mice, while the V149A mutation in H5N1 (A/Goose/Guangdong/1/96) has been shown to increase virulence in chickens [47]. Additionally, the V149A mutation reduces interferon production in chicken embryonic fibroblast cells (CEF) [48, 49].

In the case of NP protein, introducing the NP-R351K mutation promotes the effective transmission of RG-EA2, an early European avian swine virus, indicating that this minimal molecular change is essential for significantly enhancing swine transmission capabilities [9]. NP (N319K) and HA (N133D) have been described as enhancing the interaction with host factor importin-alpha, leading to increased virus replication and human-like receptor binding properties [50-53]. Another study on H5N1 has indicated that the presence of valine (V) at position 105 and lysine (K) at position 184 in the NP gene leads to increased pathogenicity in chickens [54]. Moreover, the presence of valine at position 105 (105V) may be a determining factor for avian influenza virus (AIV) adaptation from ducks to chickens [55]. Reverse genetic studies on highly pathogenic H5N1 avian influenza viruses have demonstrated that the amino acid residues 30D and 215A in the M1 protein contribute to increased pathogenicity in mice for AIV [49, 56].

**Fig. (1):**
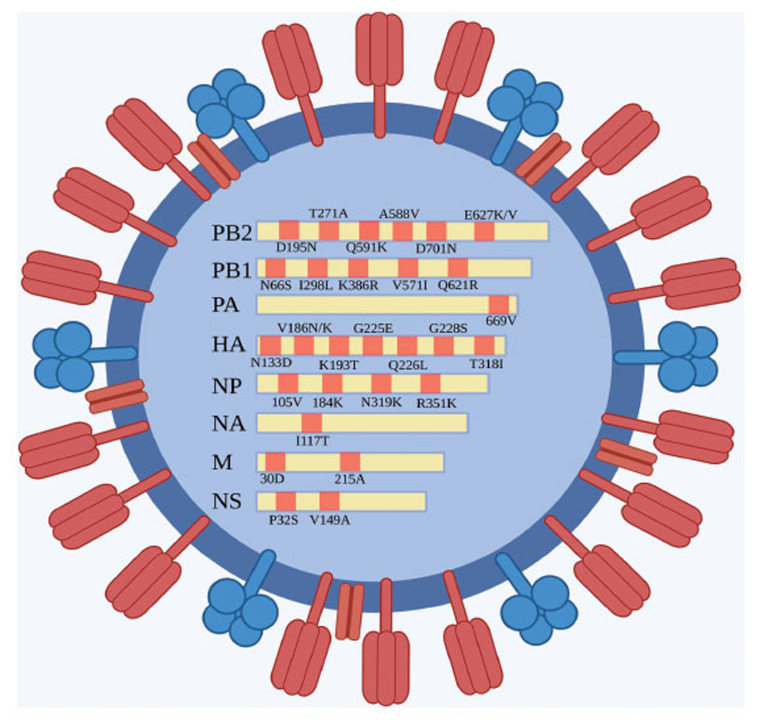
Common mutation sites of eight gene segments of the influenza virus.

In conclusion, understanding the mutations of the influenza virus is crucial for assessing its pathogenicity (Fig. **1**). In-depth research on the virus’s protein and its functions contributes to a better understanding of the mechanisms underlying influenza virus infection, laying a theoretical foundation for preventing and treating influenza and related diseases.

## 3 ANTIGEN DRIFT AND ANTIGEN SHIFT ARE TWO TYPES OF INFLUENZA VIRUS MUTATION

Antigen Drift refers to the gradual accumulation of point mutations in the influenza virus, leading to changes in surface antigens (hemagglutinin and neuraminidase). This type of mutation occurs slower and is typically predicted and targeted for adjustment in seasonal influenza vaccines. As a result of antigenic drift, varying degrees of immune protection against the same virus strain may occur within the population, gradually weakening the effectiveness of previous immunity acquired from influenza infection [57]. For example, in a study investigating two human-adapted strains of H1 hemagglutinin, it was found that residues E190 and G225 mutated to D190 and D225, altering the specific binding of HA glycoprotein to human α2,6-linked SA receptors. Specifically, HA glycoproteins containing E190/G225, E190/D225, or D190/G225 exhibited dual receptor binding specificity, while those containing D190/D225 and D190/E225 preferentially bound to human receptors. Similarly, mutations Q226L and G228S in the HA of H2 and H3 led to a shift in receptor binding preference from avian to human receptors, and in H5 HA, the Q226L mutation also resulted in a change to human receptor binding preference [58]. Furthermore, three amino acid mutations (V186N/K-K193T-G228S) in HA were reported to confer a shift in receptor specificity towards human receptors, potentially facilitating efficient transmission of type A influenza viruses within mammalian hosts [16, 59].

On the other hand, Antigen Shift involves extensive antigenic changes resulting from genetic reassortment of the influenza virus. This genetic reassortment can occur when two or more influenza virus strains infect the same host, leading to the emergence of new virus strains, and can also occur in animals. The pandemics caused by the H2N2 virus in 1957 and the H3N2 virus in 1968 are examples of genetic reassortants, deriving three (HA, NA, polymerase essential protein 1 (PB1)) or two (HA and PB1) gene segments from avian influenza viruses, along with the remaining gene segments from previously circulating human influenza viruses [1]. In 2010, a mild clinical disease-causing recombinant H1N2 influenza virus was isolated in the UK from pigs. This recombinant virus harbored a novel gene constellation, combining internal gene segments from pH1N1-origin viruses and hemagglutinin and neuraminidase gene segments from swine IAV H1N2 origin, enhancing genetic diversity of circulating strains and potentially promoting genetic changes at the human-animal interface, thus increasing the potential for generating new viruses with altered disease phenotypes or adapted to new host species. Although this virus did not exhibit increased virulence, it demonstrated high inter- and intra-species transmissibility [60-62]. In 2012, a recombinant virus carrying seven genes from pH1N1/09 and the HA gene of H5N1 could be transmitted among ferrets after acquiring the HA-N224K/Q226L/T318I mutations [63]. In 2013, the LPAIH7N9 virus acquired four amino acid insertions at the HA protein cleavage site and subsequently mutated into HPAI H7N9 in late May 2016. The HPAI H7N9 virus is further reassorted with LPAI H7N9 or H9N2 viruses, generating multiple distinct genotypes [16, 64]. In 2021, a novel H3N8 virus-causing human infection originated in chickens, representing a typical cross-species transmission event. This virus is a triple reassorting strain, possessing the H3 gene from Eurasian avian influenza viruses, the N8 gene from North American avian influenza viruses, and dynamic internal genes from H9N2 avian influenza viruses. Despite the low risk of the H3N8 virus infecting humans, with only sporadic cases reported, the virus has already acquired the ability to bind to human-type receptors. Given its potential for pandemic spread, comprehensive monitoring of the H3N8 virus in poultry populations and the environment is crucial, and close surveillance of avian-to-human transmission events is warranted [65, 66]. With the ongoing circulation of the H1N1pdm09 virus in humans, the introduction of this seasonal H1N1 virus into pigs annually has facilitated the recombination and diversification of HA and NA in local swine lineages [67]; these variations have been associated with the G155E mutation, detected in 12% of swine pdm09 genes and occurring at least 76 times in phylogenetic analysis. This amino acid plays a significant role in antigenic variation in swine H1 viruses [68].

**Fig. (2):**
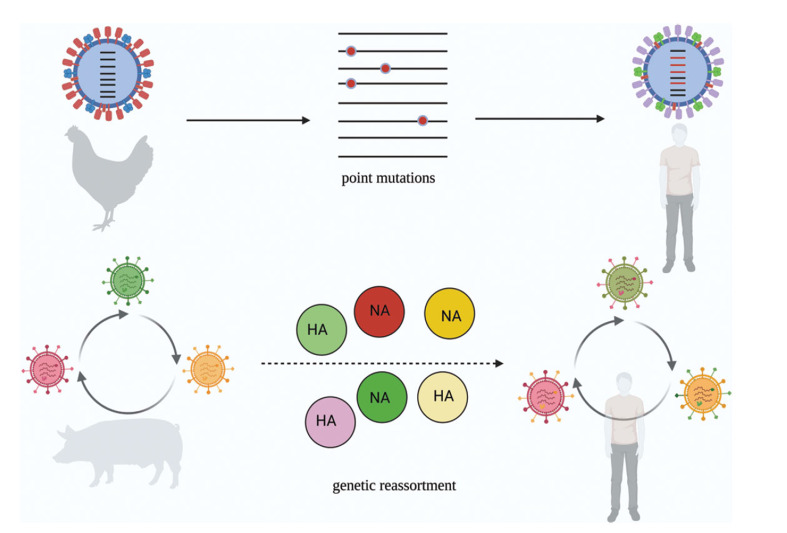
Antigen drift and antigen shift promote transmission of influenza virus interspecies.

In summary, Antigen Drift involves the gradual accumulation of point mutations in the influenza virus, leading to surface antigen changes and weakening previously acquired immunity. On the other hand, antigen shift results from the genetic reassortment of the virus, leading to extensive antigenic changes and the emergence of new virus strains with pandemic potential (Fig. **2**). Both processes highlight the ongoing challenge of influenza virus surveillance, vaccine development, and pandemic preparedness.

## 4 FACTORS INFLUENCING INFLUENZA VIRUS CROSSING SPECIES BARRIERS

Influenza viruses cross species barriers to infect humans due to two main factors: the Hemagglutinin (HA) receptor binding specificity and the ability of the viral polymerase to replicate effectively in mammalian cells. The host range of the Influenza A Virus (IAV) is determined by its ability to infect and replicate within a host. Avian Specifically, the virus must adapt its HA protein to bind to human cell receptors and ensure efficient replication in mammalian cells. Understanding and addressing these factors are essential for comprehending interspecies transmission of avian influenza viruses and developing strategies to prevent future pandemics.

## 5 HA RECEPTOR BINDING SITE

The ability of influenza viruses to cross species barriers and achieve interspecies transmission depends on overcoming replication barriers caused by species differences. The HA protein of influenza virus is responsible for binding to the surface glycoprotein structures of host cells, allowing the virus to enter and infect the host cell. There are two glycosylation receptor binding sites on the HA protein: α-2,3 and α-2,6. Human IAVs typically use long-chain α2,6-linked SA for binding, while avian IAVs use a cone-shaped topology to bind to α2,3-linked SA [1].

The α-2,3 receptor binding site refers to the region on the HA protein of the influenza virus that interacts with α-2,3 sialic acid residues on the surface of host cells. Avian influenza viruses typically use this binding site because avian respiratory tract glycoproteins contain α-2,3 sialic acid. The α-2,6 receptor binding site refers to the region on the HA protein that interacts with α-2,6 sialic acid on host cells. This binding site is predominantly used by human influenza viruses as the respiratory tract glycoproteins in humans mainly contain α-2,6 sialic acid. The type of glycosylation receptor binding sites is crucial in determining the host range and transmissibility of the influenza virus. For example, avian influenza viruses typically infect birds by binding to α-2,3 receptors on avian cell surfaces, while human influenza viruses tend to infect humans by binding to α-2,6 receptors on human cell surfaces. The replication barriers of the virus exist at the receptor binding level and cell type level, where AIV invades α-2,3 ciliated cells, and HIV invades α-2,6 non-ciliated cells [7]. So, why can avian influenza viruses occasionally infect humans? According to the available information, in the human respiratory tract, α-2,6 SA receptors are mainly expressed in upper respiratory tract ciliated cells (URT).

In contrast, α-2,3 SA receptors are mainly present in non-ciliated cells of the lower respiratory tract (LRT) and type II pneumocytes [31]. Mass spectrometry characterization of N- and o-glycan compositions in human lungs, bronchi, and nasopharynx revealed widespread presence of α-2,3 and α-2,6 glycans in the lungs and bronchi [1]. This explains why sporadic cases of the avian influenza virus can replicate and spread in the human respiratory tract [58].

Viral genomic mutations can also lead to changes in receptor specificity. This is a common occurrence. The HPAI H7N9 virus originated from the LPAI H7N9 virus, and the substitutions G186V or Q226L (H3 numbering) in the HA protein are associated with the switch in receptor specificity from avian-type (α2-3Gal) to human-type (α2-6Gal), potentially facilitating infection in mammalian hosts [31]. The single G186V mutation in the HA protein can increase the virus's affinity for human-type receptors [16]. The H10N7 virus originating from the Gull can be transmitted among minks through direct contact and aerosol transmission, showing stronger binding affinity to saliva-sialic acid with α2,3Gal and human-like α2,6Gal compared to H10 AIV of avian origin, indicating that the Q226L mutation is the leading cause of receptor binding specificity change [69, 70]. Although all wild bird AIVs in this study showed potent receptor binding affinity to prototype α2,3-s, 11 strains (32%) of H3, H6, H8, H9, H11, and H12 HA subtypes showed significant binding to α2,6-s, suggesting their potential ability to infect mammals due to the lack of necessary molecular markers for receptor binding preference [71]. The HA protein of H10N3 has receptor binding sites with 226Q and 228S, indicating dual binding specificity for avian-like and human-like receptors [18]. Like AIV, Equine IV HA strongly prefers binding to terminal sialic acid α2,3Gal, unlike human influenza [72].

The study found that if there is low affinity but a high density of avian-type receptors present, even if human-type receptors cannot directly bind to avian influenza viruses effectively, they can still enhance the binding and entry of avian influenza viruses into cells. In other words, even if human-type receptors cannot bind directly to avian influenza viruses, high-density avian-type receptors on the cell surface can indirectly facilitate avian influenza viruses' binding and entry process. This suggests that interactions and synergistic effects between different types of receptors may have important implications for the virus binding and infection process [73].

In addition to the receptor binding specificity mediated by HA discussed above, a critical factor in viral pathogenicity is the presence of multiple essential amino acids at the HA cleavage site (MBCSs). HPAIVs typically encode MBCSs at the HA cleavage site [74, 75], while HAs from mammalian viruses or LPAIVs usually encode only one essential residue at their cleavage site. Multiple basic residues allow HA to be cleaved by enzymes expressed in various tissues, whereas a cleavage site with a single essential residue can only be cleaved by enzymes in the respiratory or intestinal tract. As hemagglutinin cleavage is essential for viral infectivity, multiple primary cleavage sites expand the virus's tissue range and are closely associated with systemic spread, transmissibility, and increased virulence in birds and mammals [31, 76].

Understanding the preferences of different influenza viruses for different glycosylation receptors is crucial in better understanding their transmission pathways and potential risks of interspecies transmission. This has important implications for monitoring, preventing, and controlling influenza viruses.

## 6 MICRORNAS, AS A SPECIES BARRIER, LIMIT THE JUMPING OF INFLUENZA VIRUS

MicroRNAs (miRNAs) are a class of short, non-coding RNA molecules typically composed of 20 to 25 nucleotides. They play crucial roles in regulating gene expression and are involved in various biological processes such as cell proliferation, differentiation, apoptosis, and immune responses.

The interaction between miRNAs and influenza viruses is complex. Upon infecting host cells, influenza viruses can disrupt the expression and function of host cell miRNAs, thereby altering the host cell's gene expression profile to facilitate virus replication and infection. Conversely, host cell miRNAs can also target the influenza virus genome or gene products, affecting viral replication, transcription, translation, and assembly processes.

Studies have shown that certain miRNAs can inhibit influenza virus infection by suppressing host genes involved in virus replication. For example, in pigs, miRNA sssc-miR-221-3p and ssc-miR-222 target the viral genome, inhibiting the expression of the anti-apoptotic protein HMBOX1 and inducing cell apoptosis, thereby indirectly suppressing Avian Influenza Virus (AIV) infection and replication. Additionally, miRNAs can modulate immune response-related gene expression, influencing the host's immune reaction to influenza viruses [77]. Furthermore, miRNAs like miRNA-323, miRNA-491, and miRNA-654 have been found to bind to the PB1 gene, inhibiting H1N1 influenza A virus replication by degrading viral mRNA and downregulating PB1 expression [78]. Similarly, miR-324-5p targets the PB2 gene of the H5N1 highly pathogenic avian influenza virus (HPAIV), the negative regulator of the interferon pathway, CUEDC2, to suppress viral replication [79]. miR-584-5p and miR-1249 target the PB2 gene of the virus and suppress its replication [80]. Another study found that miR-3145 inhibits influenza virus replication by targeting and silencing the PB1 gene [81].

**Fig. (3):**
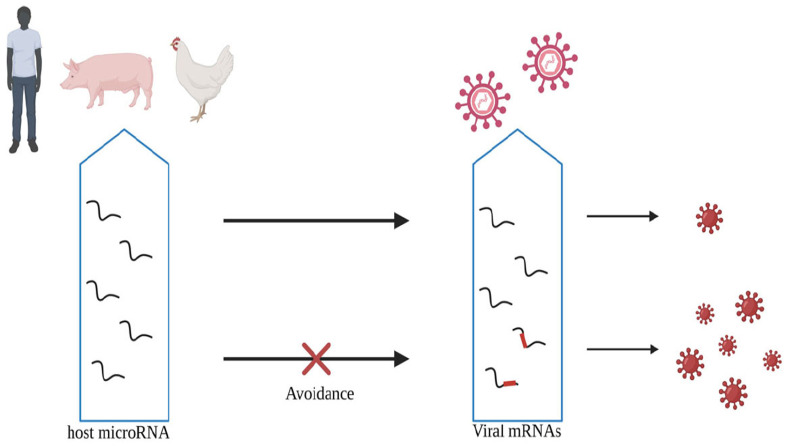
Host microRNAs can specifically recognize viral mRNAs, inhibiting viral replication and reducing pathogenicity. When the viral genome mutates to evade recognition by host microRNAs, it can promote viral replication and enhance pathogenicity. This interaction between host microRNAs and viral mRNA plays a crucial role in the host's defense against viral infections and highlights the dynamic interplay between viruses and their hosts at the molecular level.

On the other hand, microRNA-144 impairs the host cell's ability to control the replication of various viruses, including influenza virus, Yersinia pestis, and vesicular stomatitis virus. It inhibits the expression of TRAF6, which alters the cell's antiviral transcription landscape regulated by the transcription factor IRF7, resulting in reduced antiviral response [82]. Furthermore, avoided recognition by host microRNAs through amino acid substitutions or deletions in viral gene sequences can enhance the pathogenicity of the virus. In a study on low pathogenicity H9N2 adaptation in mice, multiple amino acid substitutions and evasion of host microRNA recognition led to increased virulence, fatal infection, excessive induction of pro-inflammatory cytokines, and the onset of a “cytokine storm” (Fig. **3**) [83]. Interestingly, the PB2 627(E-K) mutation located on the recognition sites of mmu-miR-1904. MicroRNAs also exhibit dual functionality. For instance, miRNA-485 has been found to regulate the antiviral immune response by targeting RIG-1, a cytoplasmic sensor of viral RNA. When the viral abundance is low, miRNA-485 binds to the 3'UTR of RIG-1 mRNA, leading to its degradation and increased viral replication.

Conversely, when the viral abundance is high, miRNA-485 binds to the viral PB1 gene, decreasing viral replication [84]. Targeting the binding sites of miRNAs or using miRNA inhibitors to silence endogenous miRNAs that promote virus development could enhance the expression of target genes, which is beneficial for human health. Promising progress has been made for tuberculosis [85, 86]. Additionally, virus-encoded miRNAs (vmiRNAs) have been discovered in human and other mammalian host cells during viral infections. VmiRNAs significantly impact viral replication and disease progression by regulating the expression of both viral and host cell mRNAs through direct cleavage or translational suppression *via* partial complementary base pairing [85]. However, until now, no influenza-encoded microRNAs have been identified.

MicroRNAs also regulate gut microbiota composition and its metabolites, maintaining a stable balance in the gut microbial community [87]. Targeting gut bacteria with specific miRNAs has been explored in treating colitis in mice [88]. MiR-181b-5p transplantation inhibits M1 macrophage polarization and promotes M2 polarization, reducing inflammation levels during chronic colitis's acute remission phases. MiR-200b-3p interacts with bacteria and regulates the composition of the microbial community, contributing to intestinal barrier integrity and homeostasis [89]. Considering microbiota could regulate the generation of virus-specific T cells and antibody responses following respiratory influenza virus infection [90]. It is possible that microRNA-microbiota interaction could modulate the host immune response to influenza virus infection.

**Fig. (4):**
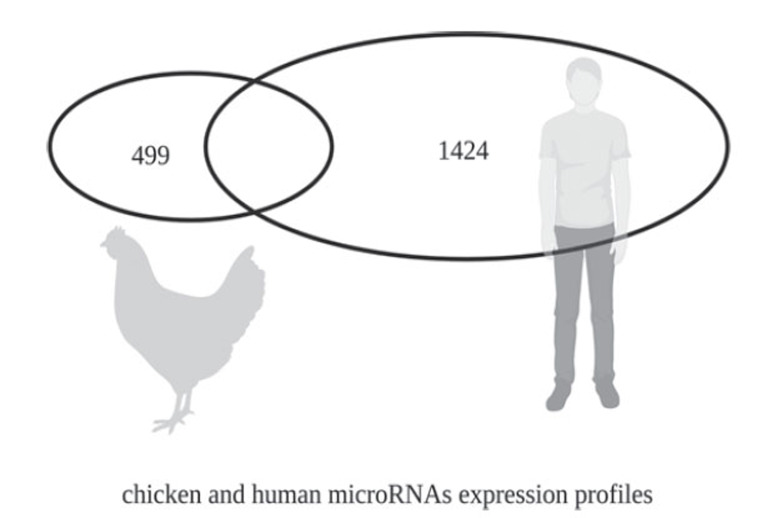
Chicken and human microRNA expression profiles.

**Fig. (5):**
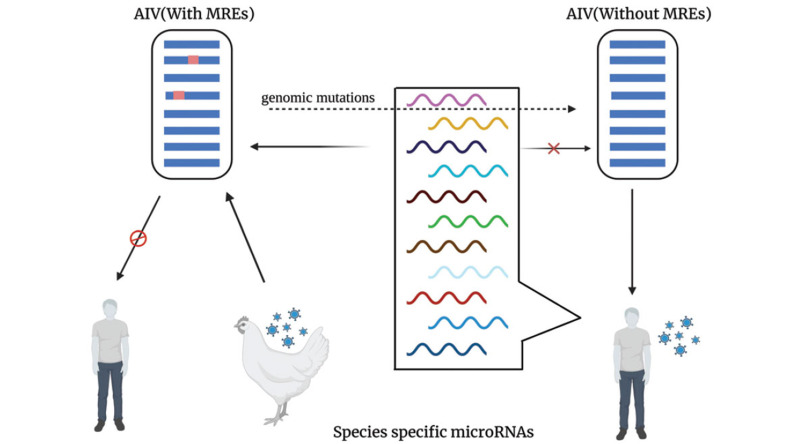
Proposed mechanism of species-specific microRNAs as a barrier determines AIV interspecies jumping.

Gene mutations allow influenza viruses to cross the species barrier and infect humans, leading to new outbreaks. microRNAs play an indispensable role in this process. Although the expression profiles of many species' microRNAs have been sequenced, many microRNAs are species-specific despite the conservation of many microRNAs across species. For example, chickens express 926 microRNAs while humans express 2300 microRNAs [91, 92], with only a subset of microRNAs being shared between the two species (Fig. **4**). If species-specifically expressed microRNAs act as inter-species barriers, they play a crucial role in determining the adaptation of avian influenza viruses to new hosts. The evolution of avian influenza viruses primarily occurs in chickens, with the viral genome possibly containing human-specifically expressed microRNA Recognition Elements (MREs). Due to the presence of these MREs, the virus cannot effectively infect humans. However, genomic mutations could eliminate the potential human-specific MREs under specific circumstances. This allows the virus to escape the inhibitory effect of microRNAs expressed explicitly in humans, thereby acquiring the capability to infect humans (Fig. **5**).

In conclusion, miRNAs play a crucial role in the interaction between hosts and pathogens in the host's immune response to microorganisms. They can potentially be targeted for therapeutic interventions by silencing detrimental target genes using miRNA-induced silencing complexes that bind to specific gene sequences or by inhibiting endogenous miRNAs, favoring virus development. Furthermore, miRNAs regulate the gut microbiota composition and metabolites, contributing to intestinal homeostasis. Understanding the mechanisms underlying miRNA-mediated regulation of viral replication and host immune responses can provide valuable insights for developing novel antiviral strategies.

## 7 INFLUENZA AND HEALTH

It is crucial to recognize that avoiding poultry alone may not prevent the spread of avian influenza. Research has shown that dogs and cats possess α-2,3 sialic acid receptors in their upper and lower respiratory tract epithelium, making direct transmission of avian influenza subtypes from poultry to dogs or cats possible. Molecular analysis reveals that many H3N2 canine isolates exhibit at least two mutations in the HA protein (Ser159Asn and Trp222Leu), which could facilitate the transmission of H3N2 influenza viruses from birds to dogs, enabling both intra- and interspecies transmission [93], Lectin histochemical analysis indicates that dogs and cats can be co-infected or sequentially infected with avian influenza and mammalian influenza viruses, potentially leading to the generation of new viral genome combinations, increasing the likelihood of endemicity and pandemics [94]. Notably, the spread of H6N8 among Antarctic bird populations and its interspecies transmission in sub-Antarctic regions underscore the complex dynamics of influenza virus transmission [95]. Genomic mutations in influenza viruses have diverse impacts on human health. Firstly, mutations can confer antiviral resistance, complicating treatment and control strategies. In 2022, two confirmed cases of human infection with the H3N8 avian influenza virus in China were reported, demonstrating a preference for binding to human receptors and possessing the PB2-E627K amino acid substitution critical for airborne transmission. Even individuals vaccinated against human H3N2 viruses may lack immunity to emerging H3N8 avian influenza viruses adapted to mammals, increasing susceptibility to epidemic or pandemic threats [12]. Secondly, genomic mutations contribute significantly to seasonal variations and large-scale influenza outbreaks. The emergence of new antigenic properties due to viral mutations reduces population immunity to novel influenza strains, leading to vaccine ineffectiveness or decreased protective efficacy. Furthermore, genomic mutations in influenza viruses can enhance their transmissibility, facilitating wider dissemination within populations and exacerbating epidemic outbreaks.

## 8 PREVENTION AND CONTROL

Practical strategies for preventing and controlling the impact of influenza virus genomic mutations on human health are crucial. Vaccine development and usage play a significant role in influenza prevention. One method to generate a universal influenza vaccine involves DNA shuffling, which creates recombinant forms of HA proteins by integrating diverse epitopes into the HA protein. The resulting chimeric HA antigen can induce broad immunity against genetically diverse influenza viruses circulating in pigs and humans [96]. PIV-5, an effective vaccine vector with constructs like PIV5-113, can protect mice and pigs from viral attacks expressing parental HAs. Expanding protective immunity against diverse gene-expressed H1 influenza viruses further enhances this approach [97]. The specificity of porcine monoclonal antibodies towards H1N1pdm09 HA closely resembles that of known human monoclonal antibodies targeting the same. These antibodies recognize two significant sites, Sa (residues 160 and 163) and Ca (residue 130), providing a good model for monoclonal therapy in humans [98, 99]. A respiratory organoid culture system derived from bat species has been developed, offering a promising tool to analyze viral infectivity and transmission dynamics between bats and other species, aiding in understanding cross-species virus spread [100, 101]. However, due to genetic mutations in the influenza virus, the efficacy of vaccines may be limited. Therefore, regular updates to vaccine strains are crucial for enhancing vaccine protection. Additionally, close monitoring of genomic variations and transmission patterns of influenza viruses, along with timely isolation and treatment measures, are vital for controlling the spread of the disease. Although avian-origin H7Nx LPAIVs lack a critical mammalian-adaptive substitution like PB2 E627K, the similarity in mortality and morbidity rates between H7N6 and H7N9 viruses in BALB/c mice suggests potential inter-species transmission. These findings underscore the need to intensify efforts to monitor the epidemiology, evolution, and risks of the zoonotic spread of avian-origin H7Nx viruses [102, 103]. Recognizing that poultry contributes to the gradual but steady expansion of highly pathogenic avian influenza [104], while wild birds such as geese, swans, gulls, and ducks facilitate rapid but sporadic dissemination through distinct pathways, is crucial for global surveillance and epidemic prediction [105, 106].

## CONCLUSION

Genomic mutations in influenza viruses are significant factors leading to interspecies transmission and affecting human health [107]. A comprehensive understanding and study of the types and mechanisms of genomic mutations in influenza viruses can effectively guide preventive and control measures against influenza. Future research should focus on monitoring genomic mutations, vaccine development, and optimization of control strategies to enhance human resistance to influenza and reduce disease transmission.

## LIST OF ABBREVIATIONS

AIV: Avian Influenza Virus
CEF: Chicken Embryonic Fibroblast Cells
HA: Hemagglutinin
HPAI: Highly Pathogenic Avian Influenza
HPAIV: Highly Pathogenic Avian Influenza Virus
HPIV: Highly Pathogenic Influenza Viruses
IAV: Influenza A Virus
LPAI: Low Pathogenic Avian Influenza
LPIV: Low Pathogenic Influenza Virus subtypes
MBS: Membrane-binding Site
miRNAs: MicroRNAs
MREs: microRNA Recognition Elements
NA: Neuraminidase
vmiRNAs: virus-encoded miRNAs
